# The synergy of metal–organic frameworks and biomaterials for bone tissue engineering: recent advances, challenges, and future recommendations

**DOI:** 10.1039/d5na00279f

**Published:** 2025-07-28

**Authors:** Luan Minh Nguyen, Yufeng Wang, Giao Thuy Quynh Vu, Qui Thanh Hoai Ta, Dieu Linh Tran, Ngoc Hoi Nguyen, Thuan Van Tran, Chao Zhang, Dai Hai Nguyen

**Affiliations:** a Institute of Advanced Technology, Vietnam Academy of Science and Technology 1B TL29 Street, An Phu Dong Ward Ho Chi Minh City 700000 Vietnam nguyendaihai0511@gmail.com nguyendaihai@iat.vast; b Graduate University of Science and Technology, Vietnam Academy of Science and Technology 18 Hoang Quoc Viet Street, Nghia Do Ward Hanoi 100000 Vietnam; c State Key Laboratory for Modification of Chemical Fibers and Polymer Materials, College of Materials Science and Engineering, Donghua University Shanghai 201620 PR China czhang@dhu.edu.cn; d Institute of Applied Technology and Sustainable Development, Nguyen Tat Thanh University 298-300A Nguyen Tat Thanh, Xom Chieu Ward Ho Chi Minh City 755414 Vietnam

## Abstract

There has recently been a noticeable increase in the prevalence of bone-related conditions, including osteoarthritis, arthritis, fractures, bone cancer, and infections, thereby creating an urgent demand for advanced biomaterials in regenerative medicine. Among emerging candidates, metal–organic frameworks (MOFs), with their large surface area, tunable porosity, and inherent bioactivity, have demonstrated considerable potential in bone tissue engineering. Initially, research focused on pristine MOFs as bioactive scaffolds or drug delivery vehicles due to their capacity for controlled encapsulation and release of therapeutic agents. However, issues such as poor stability, potential toxicity, and limited mechanical strength have driven the development of MOF-based composites. By incorporating MOFs into hydrogels, electrospun fibers, biocements, and three-dimensional scaffolds, researchers have improved biocompatibility, enhanced structural integrity, and achieved synergistic effects on bone regeneration. Consequently, these composites offer multifunctional platforms that simultaneously provide mechanical support, local drug delivery, and osteoinductive cues. This review highlights recent advances in the field, analyzes key limitations, and emphasizes the need for systematic strategies in design, synthesis, and evaluation. Furthermore, the integration of computational modeling and machine learning is proposed as a promising direction for optimizing material performance and accelerating clinical translation. Ultimately, interdisciplinary collaboration will be essential to realize the full potential of next-generation MOF-based composites in bone repair and regenerative therapies.

## Abstract 2

This review provides an overview of recent progress, challenges, and future directions in the application of MOFs and MOF-based composites for bone tissue engineering.

## Graphical abstract

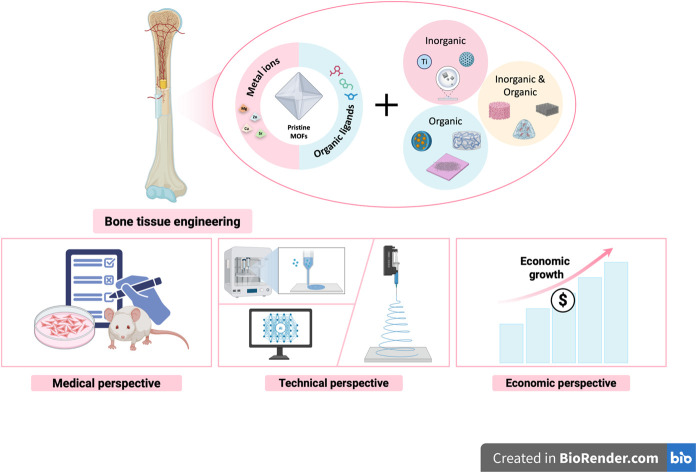

## 1. Introduction

Bones are an important body component that plays a vital role in providing structural support to the entire body, producing blood cells, storing minerals, and protecting internal organs and the nervous system.^1^ However, various bone-related diseases, *e.g.*, osteoarthritis, arthritis, bone fractures, bone cancer, and bone infections, have recently increased steadily.^2,3^ Based on statistical data, the incidence of bone fractures reported in 2019 exceeded 455 million cases, representing a notable rise of 178 million cases compared to the number of cases in 1990.^4^ In 2021, several studies reported that China exhibited a significant prevalence of over 6 million cases of bone and joint diseases with an estimated 4 million individuals necessitating immediate medical intervention, specifically bone grafts and scaffolds based on biomaterials.^5^ In the United States and Europe, bone-related issues are projected to increase by 30% (from 2005 to 2025) and 28% (from 2010 to 2025), respectively.^6,7^ This scenario arises from subjective and objective factors, encompassing population aging, occupational accidents, postoperative complications, and cancer cell metastasis.^8–10^ Bone diseases, regardless of the cause, have a profound impact on human health and quality of life.^11,12^ Indeed, bone-related issues, *e.g.*, fractures, may lead to decreased productivity, absenteeism, and disability, thus causing a health barrier for patients, along with a financial burden and an impact on the development of national economic potential.^13,14^

Based on these aspects, biomaterials capable of promoting bone growth, recovery, and regeneration have been elaborated. Titanium alloys, for example, have been built into implants such as prosthetic joints, screws, and plates that are used to immobilize, link, and accelerate bone repair.^15–17^ Specifically, titanium-based implants with good load-bearing capacity, wear resistance, and biological inertness have been widely used in clinical bone implantation.^18,19^ In addition, organically derived biomedical materials (*e.g.*, collagen, chitosan, and hyaluronic acid) have been developed into hydrogel systems, which are known to provide moisture and increase adhesion, bone cell proliferation, and differentiation.^20–23^ On the other hand, biocements, including calcium phosphate, tricalcium phosphate, magnesium phosphate, and calcium sulfate hemihydrate, are used to fill gaps or scaffolds in bone surgery.^24–28^ With the advancements in biomedical technology, the integration of inorganic and organic materials has become an integral part of the fabrication of three-dimensional printed constructs. In this process, biological scaffolds are not only shaped with high precision conforming to computer-aided designs, but are also expected to exhibit improved biological properties, mechanical durability, and replaceability of natural bones.^29–32^ Nevertheless, to address the increasing complexity of bone-related pathogenic variants, biomaterials need to be more flexible, intelligent, and multifunctional. As a result, there is a need to explore advanced materials to integrate with those that have achieved remarkable results in bone tissue engineering, aiming to create versatile composite materials with desired therapeutic efficiencies.

Metal–organic frameworks (MOFs) represent one of the promising advanced materials, constructed from two primary components, namely metal ions/clusters and organic ligands.^33–36^ Noteworthy characteristics of MOFs include their large specific surface area, diverse porous structures, and flexible, tunable frameworks based on both inorganic and organic constituents.^37–39^ In addition, some families of MOFs, such as zeolitic imidazolate frameworks (ZIFs), Universitetet i Oslos (UiOs), and MILs (Lavoisier Laboratory), also possess high thermal, chemical, and mechanical stability.^40–43^ Regarding applications, MOFs have garnered significant research attention in the fields of environment, energy, and biomedicine.^44–46^ Indeed, MOFs have been discovered with many potential applications in bone tissue engineering throughout recent years. This may arise from the structure of MOFs, which contain trace elements (*e.g.*, zinc, magnesium, calcium, and strontium) that could promote the regeneration and differentiation of bone cells.^47,48^ In addition, organic ligands derived from amino acids, nucleobases, and vitamins can be absorbed by the body, thereby limiting the toxicity accumulated during prolonged treatment.^49–51^ Effective antibacterial properties were also discovered in some MOFs, such as Zn-MOFs, Cu-MOFs, Co-MOFs, and Fe-MOFs.^52–54^ Nano-sized MOFs have been demonstrated as potential candidates for efficient storage and transport of bioactive agents (*e.g.*, drugs, enzymes, and DNA) within the physiological system of the body. Besides, MOFs can be easily modified to be responsive to stimuli such as pH, near-infrared (NIR) light, and enzymes for targeted pharmacological applications.^55–57^

In general, there is a significant increase in the number of studies on MOFs in bone tissue engineering. Therefore, the systematic collation of literature on this subject holds considerable significance. Specifically, we reviewed studies on the primitive applications of MOFs, followed by their integration with inorganic and organic biomaterials for treating bone injuries. Within this narrative, the pivotal roles played by therapeutic elements, including metal ions, organic ligands, and drugs, acting as active pharmaceutical ingredients, were elucidated. Additionally, the positive contributions of MOFs in bone regeneration, infection prevention, inflammation reduction, and malignant bone tumor treatment were highlighted. However, the application of MOFs in clinical settings still faces numerous challenges related to molecular building blocks, physiological properties, biological properties, and synthesis methods. As a result, personal perspectives were proposed to clarify ambiguities and the emerging applications of MOFs in bone tissue engineering. Furthermore, a thorough examination of medical, technical, and economic aspects was conducted to ensure that the integration of MOFs into bone tissue engineering not only benefits patient outcomes but also enhances the healthcare industry as a whole.

## 2. Pristine MOFs and MOFs loaded with bioactive agents for bone tissue engineering

### 2.1. Pristine MOFs

As previously discussed, pristine MOFs consist of metal ions or clusters and organic ligands, exhibiting low levels of biological toxicity and stimulating cell development at appropriate concentrations.^59,60^ Moreover, the metal ions (*e.g.*, Ca^2+^, Sr^2+^, Mg^2+^, and Zn^2+^) incorporated into the structure of MOFs can serve as trace elements that support bone growth.^61,62^ Hence, the application of pristine MOFs in bone tissue engineering has recently attracted many studies (Table 1). For instance, Matlinska and co-workers^63^ developed bioMOF systems by incorporating alkaline earth metal ions (Ca^2+^ and Sr^2+^) together with a *p*-xylylenebisphosphonate ligand, resulting in the formation of SrCaPAEM, CaPAEM, and SrPAEM for potential application in osteoporosis treatment. These bioMOFs serve as dual sources of therapeutic metal ions (Ca^2+^ and Sr^2+^) and bisphosphonate molecules, contributing to the maintenance of normal bone density. In addition, their interaction with bovine serum albumin improves protein adsorption, thereby promoting osteoblast proliferation and facilitating bone regeneration.

**Table 1:** Pristine MOFs and MOFs loaded with bioactive agents for bone tissue engineering^a^

MOF	Bioactive	MOF-based biomaterial	Properties	Ref.
**Pristine MOF**				
SrPAEM bioMOF	—	SrPAEM bioMOF	Biocompatibility and bone mineralization	63
CaPAEM bioMOF	—	CaPAEM bioMOF	Biocompatibility and bone mineralization	63
SrCaPAEM bioMOF	—	SrCaPAEM bioMOF	Biocompatibility and bone mineralization	63
Cu l-Asp bioMOF	—	Cu l-Asp bioMOF	Osteogenesis and angiogenesis	58
MgCu-MOF74	—	MgCu-MOF74	Osteogenesis and antibacterial properties	64

**MOFs loaded with bioactive agents**				
Mg-MOF-74	Ket	Ket@Mg-MOF-74	Osteogenesis and anti-inflammatory properties	69
ZIF-8	MicroRNAs	MicroRNAs@ZIF-8	Osteogenesis and angiogenesis	70
ZIF-8	RIS	RIS@ZIF-8	Osteogenesis	76
ZIF-8	7,8-DHF	7,8-DHF@ZIF-8	Osteogenesis and angiogenesis	75
ZIF-8	CEL	CEL@ZIF-8	Osteogenesis and antibacterial and anti-inflammatory properties	77

a Ket: ketoprofen; MicroRNAs: proangiogenic miR-21 and pro-osteogenic miR-5106; RIS: risedronate; 7,8-DHF: 7,8-dihydroxyflavone; CEL: celecoxib.

**Fig. 1:**
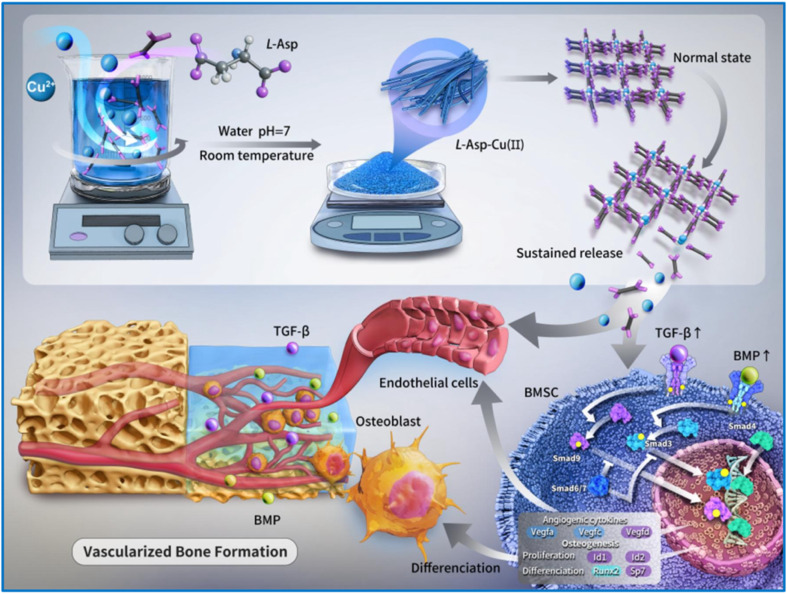
Schematic illustration of the fabrication and mechanism of l-Asp-Cu(ii) bioMOF for vascularized bone regeneration. Cul-Asp is synthesized through the coordination of Cu^2+^ ions and l-aspartic acid, enabling the sustained release of bioactive Cu^2+^. These ions activate the TGF-β/BMP signaling pathway, promoting neovascularization and accelerating bone tissue regeneration. This figure has been reproduced from ref. 58 with permission from Elsevier, copyright 2025.

In another study, Liu *et al.*^64^ conducted a comparative investigation on bone regeneration using Mg-MOF-74 and MgCu-MOF-74. *In vitro* assessment indicated that both types of MOF-74 could facilitate the growth of human osteogenic sarcoma cells (SaOS-2) for 5 days, in which MgCu-MOF-74 showed dominant activity. This is attributed to the synergistic effect of Mg^2+^ and Cu^2+^ ions in enhancing the adhesion, proliferation, and differentiation of bone cells. Apart from promoting cell growth, Cu^2+^ in MgCu-MOF-74 has proven to offer significant antimicrobial efficacy in clinical applications for minimizing implant infections and postoperative recovery. Vascularized bone regeneration, on the other hand, is another crucial process in bone repair that has been extensively explored in recent studies. Zhang and co-workers^65^ introduced a novel l-Asp-Cu(ii) bioMOF, which was constructed through the coordination bonding between l-aspartic acid (l-Asp) and Cu^2+^ ions. The remarkable results from *in vitro* and *in vivo* studies demonstrated that bioMOF, with its sustained release of bioactive Cu^2+^ ions, effectively activated the TGF-β/BMP signaling pathway, thereby promoting neovascularization and accelerating bone regeneration at the defect site (Fig. 1).

### 2.2. MOFs loaded with bioactive agents

**Fig. 2:**
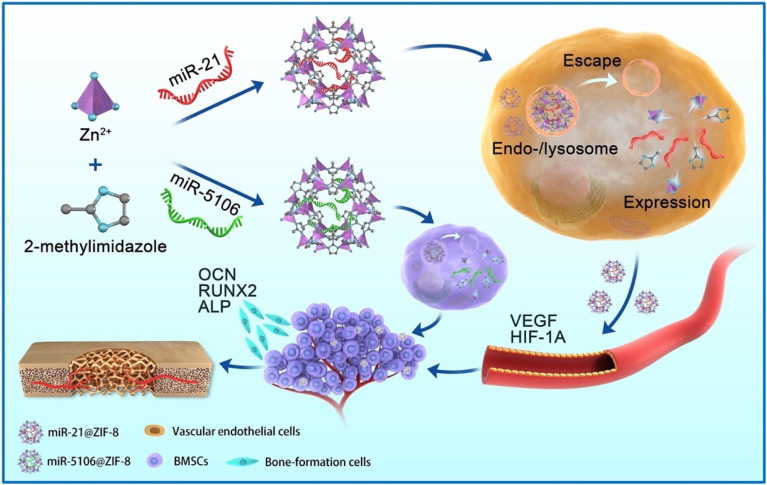
Schematic representation of miR@ZIF-8 nanocomposites for the delivery of proangiogenic microR-21 and pro-osteogenic microR-5106. These nanocomposites enable efficient cellular internalization and microRNA release, which upregulate angiogenic (VEGF and HIF-1A) and osteogenic (ALP, OCN, and RUNX2) genes, thereby promoting vascularization and bone regeneration. This figure has been reproduced from ref. 70 with permission from Elsevier, copyright 2022.

To improve the effectiveness of bone disease treatments, pristine MOFs have been combined with therapeutic agents, including drugs, microRNAs, metal ions, and flavonoid glycosides. In this role, MOFs act as carriers that encapsulate, transport, and control the release of these agents, thereby providing essential factors for bone repair and recovery.^66–68^ For example, Ge *et al.*^69^ created a drug delivery system Ket@Mg-MOF-74 by loading the drug ketoprofen (Ket) into the structure of Mg-MOF-74. This system showed the ability to encapsulate and release a high drug payload with promising results. Moreover, the qPCR results from the MG63 osteoblastic model over 5 days indicated that Ket@Mg-MOF-74 reduced the expression of pain-related genes (COX2) and inflammatory factors (TNF-α, IL-1β, and IL-6), while also stimulating osteogenic genes (BMP2, RUNX2, and ALP). Feng *et al.*^70^ developed miR@ZIF-8 nanocomposites *via* a one-pot synthesis to deliver proangiogenic (microR-21) and pro-osteogenic (microR-5106) microRNAs. With an average size of 242 nm, these nanocomposites enabled microRNA release within acidic endo-/lysosomes, effectively upregulating angiogenic (VEGF and HIF-1A) and osteogenic (ALP, OCN, and Runx2) genes, thus facilitating vascularized bone regeneration (Fig. 2).

In addition, the process of bone formation can be activated through the interaction between brain-derived neurotrophic factor (BDNF) and receptor tyrosine kinase B (TrkB). This process generates signal pathways in cells for the regulation of differentiation and bone formation.^71–73^ However, BDNF has an inherent weakness in terms of short half-life and poor distribution efficiency.^73,74^ To overcome this barrier, Sun and co-workers^75^ reported that the plant-derived flavonoid 7,8-dihydroxyflavone (7,8-DHF) could be a potential solution to replace BDNF with similar biological effects. Furthermore, 7,8-DHF is contained in the ZIF-8 structure (7,8-DHF@ZIF-8) to strengthen sustainability in physiological environments. The *in vitro* results indicated that 7,8-DHF@ZIF-8 at concentrations lower than 50 mg L^−1^ facilitated angiogenesis and bone formation.

## 3. Integration of MOFs with organic biomaterials for bone tissue engineering

### 3.1. Targeted and controlled drug delivery

However, despite the advancements discussed in Section 2, MOFs still present several inherent limitations, including suboptimal targeting capability, inadequate control over the release of bioactive agents, and insufficient biocompatibility in physiological environments. To address these challenges, considerable research efforts have focused on encapsulating MOFs with biocompatible polymers or active targeting agents to form core–shell structures, thereby enhancing their stability, selectivity, and controlled release behavior (Table 2).

**Table 2:** MOFs incorporated with organic biomaterials for bone tissue engineering^a^

MOF	Biomaterial	Bioactive	MOF-based biomaterial	Stimuli	Properties	Ref.
**Targeted and controlled drug delivery**						
ZIF-8	SCM	—	SCM coated ZIF-8	—	Osteogenesis	100
ZIF-8	Gel	SIM and Aln	Aln/SIM@ZIF-8/Gel	—	Bone-targeted drug delivery	101
ZIF-8	PVP	ZOL and DOX	PVP coated ZOL@DOX@ZIF-8	pH	Bone-targeted drug delivery	102
ZIF-8	PVP	ZOL and BSA	PVP coated ZOL@BSA@ZIF-8	pH	Bone-targeted drug delivery	102
ZIF-8	FPD and MV	MTX	FPD/MV coated MTX@ZIF-8	pH	Bone-targeted drug delivery and anti-inflammatory properties	103
ZIF-8	HA	CUR and Aln	HA/Aln coated CUR@ZIF-8	pH	Bone tumor-targeted drug delivery and anti-cancer properties	79
ZIF-90	PEG	5-Fu, ICG, and ZOL	ZOL-PEG coated 5-Fu/ICG@ZIF-90	pH, NIR	Bone tumor-targeted drug delivery and anti-cancer properties	80
ZIF-8	PVP	ICG, Cyt c, and ZOL	ZOL-PVP coated ICG/Cyt c@ZF-8	pH, NIR	Bone tumor-targeted drug delivery and anti-cancer properties	81
ZIF-8	ECM	DEX	ECM coated DEX@ZIF-8	—	Osteogenesis	78

**MOF-modified hydrogels**						
ZIF-8	SA	—	ZIF-8 modified SA	—	Osteogenesis	104
ZIF-8	CA and CS	—	ZIF-8 modified CA-CS	—	Osteogenesis and angiogenesis	85
ZIF-8	L-DP	—	ZIF-8 modified L-DP	—	Osteogenesis and angiogenesis	105
ZIF-8	Fibrin	—	ZIF-8 modified fibrin	—	Osteogenesis	106
CuTA bioMOF	SF	—	CuTa modified SF	—	Osteogenesis and antioxidant and antibacterial properties	86
Mg/Fe-MOF	PAA	—	Mg/Fe-MOF modified PAA	—	Osteogenesis	107
ZIF-8	GelMA	—	ZIF-8 modified GelMA	—	Osteogenesis and antibacterial properties	108
ZIF-67	GelMA and eIm	—	eIm/ZIF-67 modified GelMA	—	Osteogenesis	109
ZIF-8	PEGDA and SA	SIM	SIM@ZIF-8 modified PEGDA/SA	—	Osteogenesis	87
ZIF-8	PAM and CMC	Aln	Aln@ZIF-8 modified PAM-CMC	—	Osteogenesis	110
ZIF-8	GelMA and CMCS	CGRP	CGRP@ZIF-8 modified CMCS/GelMA	pH	Osteogenesis and angiogenesis	82

**MOF-modified fibers**						
ZIF-8	PCL and Col		ZIF-8 modified PCL/Col	—	Osteogenesis and angiogenesis	88
ZIF-8	PCL and LIG	—	ZIF-8 modified PCL/LIG	—	Osteogenesis and antioxidant and antibacterial properties	111
Zn-Cu MOF	PLLA	—	Zn-Cu MOF modified PLLA	—	Osteogenesis and antibacterial properties	112
ZIF-8	PVA, CS, and HA	—	ZIF-8 modified PVA/CH/HA	—	Osteogenesis and antibacterial properties	113
Ni-MOF	*β*-CDs	—	Ni-MOF modified *β*-CDs	—	Osteogenesis	114
ZIF-8	PCL	BMP-6	BMP-6@ZIF-8 modified PCL	—	Osteogenesis	97
ZIF-8	PG	Aln	Aln@ZIF-8 modified PG	—	Osteogenesis	115
CuBDC	PLGA	Exo	Exo@ZIF-8 modified PLGA	—	Osteogenesis and angiogenesis	96
UIO-66	CS	FOS	FOS@UIO-66 modified CS	—	Osteogenesis and antimicrobial properties	116
ZIF-8	PVA	VAN	VAN@ZIF-8 modified PVA	pH	Biocompatibility and antimicrobial properties	117
ZIF-8	CS	VAN	VAN@ZIF-8 modified CS	pH	Osteogenesis and antibacterial properties	118
ZIF-8	Gel	Phe	Phe@ZIF-8 modified Gel fiber	NIR	Osteogenesis, bone-targeted drug delivery and anticancer properties	119

**MOF-modified PEEK**						
ZIF-8	PDA/PEEK	—	ZIF-8 modified PDA/PEEK	NIR	Osteogenesis and antibacterial properties	98
ZIF-8	PDA/PEEK	SIM	SIM@ZIF-8 modified PDA/PEEK	NIR	Osteogenesis and antibacterial properties	99
Zn-Mg-MOF74	PDA/PEEK	DEX	DEX@Zn-Mg-MOF74 modified PDA/PEEK	—	Osteogenesis and antibacterial properties	89

a PVP: polyvinyl pyrrolidone; ICG: indocyanine green; Cyt c: cytochrome c; Col: collagen; SIM: simvastatin; Aln: alendronate; Gel: gelatin; ZOL: zoledronate; DOX: doxorubicin; BSA: bovine serum albumin, MV: macrophage-derived microvesicle, FPD: 1,2-distearoyl-*sn*-glycero-3-phosphoethanolamine-*N*-[folate (polyethylene glycol)-2000], MTX: methotrexate, HA: hyaluronic acid; CUR: curcumin; PEG: polyethylene glycol; 5-Fu: 5-fluorouracil, SA: sodium alginate; PEGDA: poly(ethyleneglycol) diacrylate; GelMA: gelatin methacryloyl; PAM: polyacrylamide; CMC: carboxymethylcellulose; Aln: alendronate; CA: catechol; CS: chitosan; L-DP: l-dopa amino acid/poly(vinyl alcohol); eIm: 2-ethylimidazole; PAA: poly(acrylic acid); PVA: polyvinyl alcohol; PCL: polycaprolactone; LIG: lignin; BMP-6: bone morphogenetic protein-6; PLLA: poly-l-lactic acid; PG: polycaprolactone/gelatin; Phe: phenamil; Exo: exosomes; PLGA: poly(lactic acid-*co*-glycolic acid); FOS: fosfomycin; β-CDs: β-cyclodextrins; PDA/PEEK: polydopamine modified polyetheretherketone; PVDF: polyvinylidene fluoride; SCMs: stem cell membranes; ECM: extracellular matrix; DEX: dexamethasone; SF: silk fibroin; CMCS: carboxymethyl chitosan; CGRP: calcitonin gene-related peptide.

Accordingly, Shen *et al.*^79^ illustrated a bone-targeted drug delivery system using the anti-osteoclastic drug curcumin (CUR) loaded onto pH-sensitive nanocarrier ZIF-8 and further coated it with dual-targeting ligands, hyaluronic acid (HA) and alendronate (ALN), termed CZ@HA/ALN. Leveraging the inherent pH sensitivity of nanocarrier ZIF-8, the Zn^2+^ and 2-methylimidazole bonds were disrupted by protonation in the acidic tumor environment, enabling drug release. The drug release profiles indicated that CZ@HA/ALN showed a Cur release efficiency of 52.25 ± 2.77% at pH 5.0, which was 3.3 times higher than that at pH 7.4, after 48 hours. HA and ALN, as tumor- and bone-targeting ligands, conferred cancer cell targeting ability to the CZ@HA/ALN system, as evidenced by its superior anticancer efficacy compared to free Cur. In mouse models with tibial metastases, the CZ@HA/ALN system achieved a tumor suppression rate of 51.62 ± 4.91%, compared to 18.61 ± 5.91% for direct CUR use.

Additionally, targeted drug delivery systems combining chemotherapy and photothermal therapy for bone metastasis have garnered attention. For example, Ge *et al.*^80^ employed ZIF-90 as a pH-sensitive drug carrier to co-deliver the anticancer drug 5-fluorouracil (5-Fu) and the photoactive agent indocyanine green (ICG). To improve stability and bone-targeting capability, this nanoplatform was further coated with polyethylene glycol (PEG) and zoledronic acid (ZOL), resulting in the formation of 5-Fu/ICG@ZIF-90-PEG-ZOL. As anticipated, both *in vitro* and *in vivo* studies demonstrated that 5-Fu/ICG@ZIF-90-PEG-ZOL not only enabled the controlled release of 5-Fu but also achieved efficient photothermal conversion under NIR light at the metastatic bone cancer site, thereby significantly enhancing therapeutic efficacy. In a similar approach, Jiang and coworkers^81^ also reported a ZIF-8-based nanoplatform capable of effectively inhibiting cancer cells and bone metastasis in BALB/c mouse models.

**Fig. 3:**
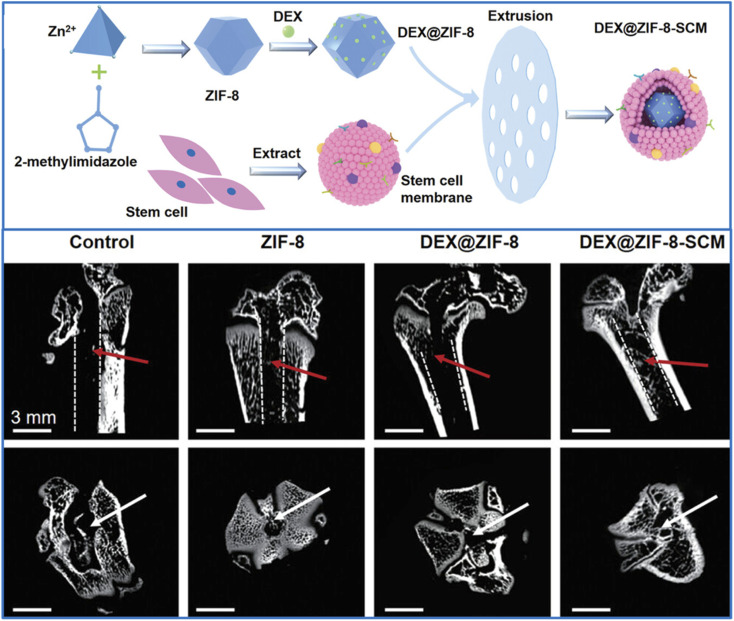
The schematic illustrates the synthesis process of DEX@ZIF-8 and DEX@ZIF-8-SCM nanomaterials. Coronal and horizontal micro-CT images of femoral defect models reveal a markedly enhanced bone regeneration capacity in the DEX@ZIF-8-SCM group compared to the control, ZIF-8, and DEX@ZIF-8 groups. This figure has been reproduced from ref. 78 with permission from John Wiley and Sons, copyright 2022.

Besides the extensive utilization of polymers, stem cell membranes (SCMs) have also been applied as coatings on the surface of MOFs to develop bioinspired targeted drug delivery systems. The notable advantages of SCMs lie in their ability to actively direct nanoparticles toward specific target cells, minimize immune responses, and prolong systemic circulation time. Moreover, SCMs can provide membrane proteins that facilitate the bone healing process. A representative study illustrating this approach is presented in Fig. 3. In this study, Liang *et al.*^78^ synthesized ZIF-8 nanoparticles loaded with dexamethasone (DEX) *via* physical adsorption, termed DEX@ZIF-8, followed by the coating of SCMs onto the nanoparticles to form DEX@ZIF-8-SCM. The effectiveness of the approach was demonstrated by the superior behavior of DEX@ZIF-8-SCM, which showed efficient cellular uptake and sustained DEX release in mesenchymal stem cells (MSCs). The results from a rat femoral defect model further confirmed that DEX@ZIF-8-SCM significantly improved bone regeneration compared to MSCs (control group), ZIF-8, and DEX@ZIF-8.

### 3.2. MOF-modified hydrogels

Based on the advancements obtained, MOFs and MOF-based drug delivery systems have also been integrated into other organic platforms. For instance, hydrogels possess excellent biocompatibility by mimicking the natural extracellular matrix (ECM) and stimulating cell proliferation.^83^ However, the use of hydrogels has many disadvantages, including low osteogenic efficiency, weak mechanical strength, and limited stability in physiological environments. These weaknesses can be addressed by leveraging the inherent strengths of MOF systems.^84^

Liu *et al.*^85^ modified a catechol-chitosan (CA-CS) hydrogel with ZIF-8 dosages of 0.6, 1.2, and 2.0 mg, respectively. Based on structural characterization analysis, ZIF-8 at a dosage of 1.2 mg was deemed suitable for developing an injectable CA-CS/Z formulation. The results of micro-CT analysis on an SD rat skull defect model showed that CA-CS/Z hydrogel possessed a bone volume/total volume ratio of 22.95% ± 2.39%, which was 1.5 times greater than that of CA-CS hydrogel and 2.7 times greater than that of the control sample. In another study, Cao and colleagues^86^ worked on MOF nanozymes from copper nanoparticles and tannic acid (CuTA), and then incorporated them with the silk fibroin (SF) to form CuTA@SF hydrogel. The CuTA@SF hydrogel had a pore size of 131.9 ± 11.10 μm and a porosity of 23.34 ± 5.70% and offered a biological framework for bone cell development. Indeed, CuTA@SF hydrogel reached promising results on models of femoral defects in New Zealand rabbits. Specifically, bone mineral density (BMD) was 0.3 g cm^−3^, bone volume/total volume (BV/TV) was 20%, trabecular thickness (Tb. Th) was 225 μm, and the trabecular number (Tb. N) was 1.05 mm^−1^.

**Fig. 4:**
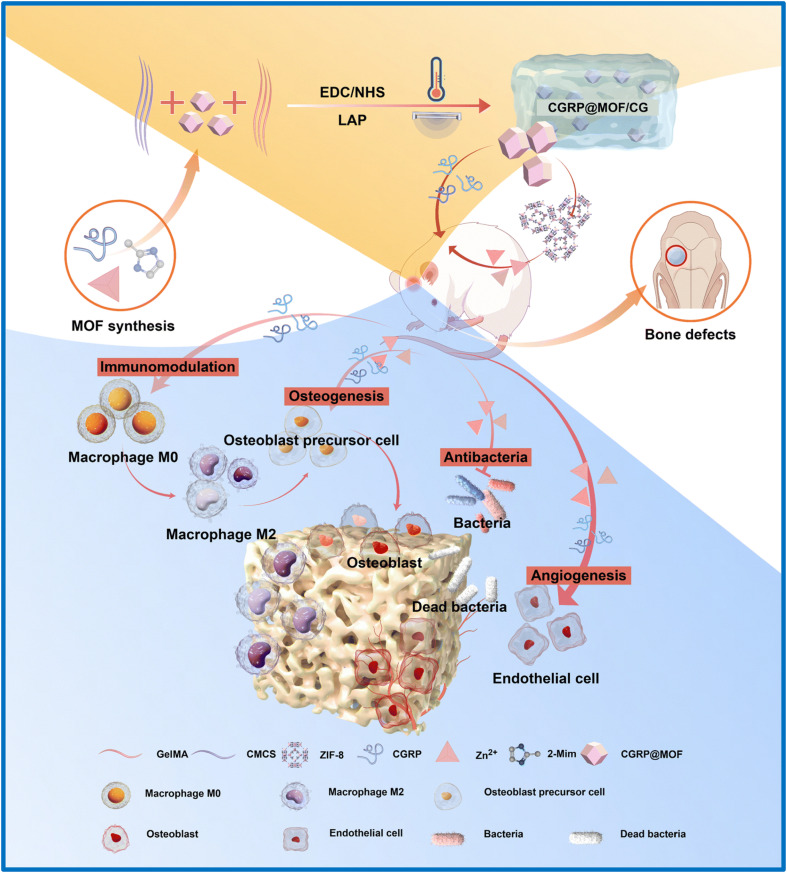
Schematic illustration of the synthesis process of the CGRP@MOF/CG hydrogel. The CGRP@MOF/CG hydrogel enables sustained release of CGRP and Zn^2+^, thereby enhancing antibacterial properties and promoting angiogenesis and osteogenesis-related factors. This figure has been reproduced from ref. 82 with permission from the Royal Society of Chemistry, copyright 2025.

On the other hand, Qiao *et al.*^87^ elevated the mechanical strength of hydrogels by developing simvastatin loaded with ZIF-8 (SIM@ZIF-8) and then dispersed it into a mixture of poly(ethylene glycol) diacrylate (PEGDA) and sodium alginate (SA) to create a nano SIM@ZIF-8/PEGDA/SA hydrogel (defined as nSZPS). As expected, the nSZPS hydrogel possesses a mechanical strength of 1 MPa and is 1.6 times more durable than PEGDA/SA hydrogel. This advancement can be attributed to the interface binding force between the PEGDA/SA polymer matrix and nano SIM@ZIF-8. The nSZPS hydrogel with sustained release of Zn^2+^ (about 6 mg L^−1^) and SIM (about 4.1 mg L^−1^) stimulated osteogenic-related genes (ALP, RUNX2, OCN, and OPN) of BMSCs after 7 days. Lou *et al.*^82^ successfully fabricated a multifunctional composite hydrogel in which calcitonin gene-related peptide (CGRP) was encapsulated within a ZIF-8 framework (CGRP@MOF) and subsequently incorporated it into a carboxymethyl chitosan–gelatin methacryloyl (CG) hydrogel matrix. The CGRP@MOF/CG hydrogel enabled the sustained release of both CGRP and Zn^2+^ ions, which promoted angiogenesis and osteogenic differentiation in both *in vitro* and *in vivo* models. Additionally, it modulated macrophage polarization toward the M2 phenotype, thereby enhancing the local immune microenvironment. Additionally, the hydrogel exhibited effective antibacterial activity against both *Staphylococcus aureus* (*S. aureus*) and *Escherichia coli* (*E. coli*), emphasizing its potential applications in bone tissue regeneration and infection control (Fig. 4).

### 3.3. MOF-modified fibers

Recently, electrospun fibers with organic components, such as polyvinyl alcohol (PVA), polycaprolactone (PCL), and poly(lactic acid-*co*-glycolic acid) (PLGA), have been extensively investigated for application in bone regeneration toward bone-damaged tissues.^90,91^ The key element of this approach can be traced to the high biocompatibility of the above components.^92,93^ Furthermore, through electrospinning, the organic components have been shaped into a micro/nano-fiber shape with interconnected pores, bearing resemblance to the ECM, which is suitable for adhesion and cell proliferation.^94^ However, one of the main challenges with conventional electrospun fibers is their inadequate supply of bone growth factors.^95^

**Fig. 5:**
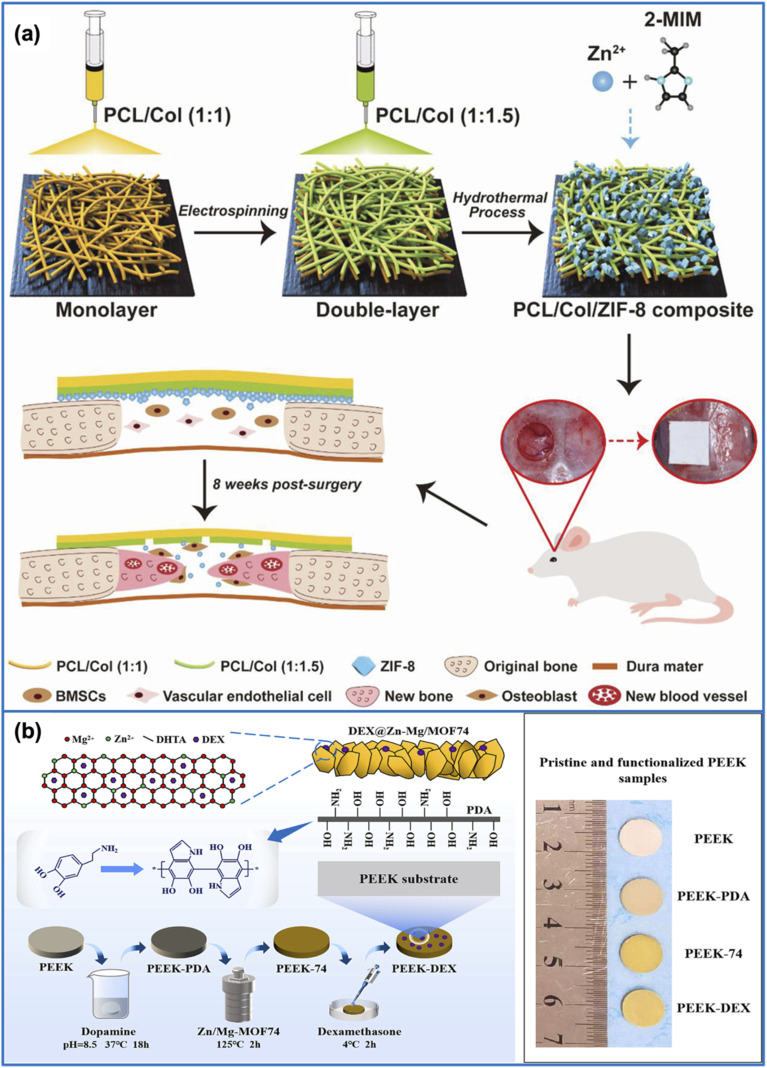
(a) Schematic illustration of the fabrication process of the PCL/Col/ZIF-8 composite membrane. The resulting composite membrane demonstrated promising potential for vascularized bone regeneration after 8 weeks of implantation in a rat calvarial defect model. This figure has been reproduced from ref. 88 with permission from John Wiley and Sons, copyright 2021. (b) Schematic illustration of the synthesis process of Zn/Mg-MOF74 coating on the surface of PEEK implants. Initially, PEEK was pretreated with PDA to increase surface adhesion. Subsequently, a layer of Zn/Mg-MOF74 was grown on the surface of PEEK-PDA *via* a hydrothermal method, forming a PEEK-74 composite. Finally, PEEK-74 was loaded with DEX to promote bone regeneration. This figure has been reproduced from ref. 89 with permission from the American Chemical Society, copyright 2021.

To address this drawback, Xue and colleagues^88^ employed ZIF-8 to modify polycaprolactone/collagen (PCL/Col) fibers. Specifically, after electrospinning and shaping into membranes, the PCL/Col fibers were directly immersed in a hydrothermal reactor containing zinc nitrate hexahydrate and 2-methylimidazole precursors to form a PCL/Col/ZIF-8 composite membrane. Both *in vitro* and *in vivo* studies demonstrated that the PCL/Col/ZIF-8 composite membrane provided a favorable microenvironment in which the sustained release of Zn^2+^ ions from the structure effectively stimulated bone tissue and blood vessel formation in a rat calvarial defect model, outperforming both PCL and Col membranes (Fig. 5a).

In another study, Xu *et al.*^96^ incorporated CuBDC-MOF directly into the PLGA solution, followed by electrospinning to fabricate PLGA/CuBDC scaffolds. Subsequently, exosomes (Exo), which are biological agents that actively promote both osteogenesis and angiogenesis, were immobilized on the surface to form multifunctional PLGA/CuBDC@Exo scaffolds. Benefiting from the presence of CuBDC-MOF, the PLGA/CuBDC@Exo scaffolds exhibited a sustained release profile, maintaining approximately 90% Exo release over 7 days compared with the faster release observed within 4 days in the PLGA scaffolds alone. This sustained release environment significantly elevated osteogenic and angiogenic expressions (*e.g.*, Ocn, ALP, Runx2, CD31, and VEGF) in *in vivo* models.

Besides, Toprak *et al.*^97^ directly embedded ZIF-8 nanoparticles loaded with bone morphogenetic protein-6 (BMP-6@ZIF-8) into the PCL solution before electrospinning to fabricate a PCL/BMP-6@ZIF-8 membrane. This composite system exhibited a high BMP-6 loading efficiency of approximately 98% and maintained a sustained release profile over 30 days. Owing to these properties, results from a Wistar rat calvarial defect model demonstrated that the PCL/BMP-6@ZIF-8 membrane achieved new bone volume formation of approximately 17%, which was 7% higher compared to the electrospun PCL membrane without BMP-6.

### 3.4. MOF-modified PEEK

Polyetheretherketone (PEEK), a high-performance polymer, has garnered considerable attention in bone tissue engineering owing to its excellent mechanical strength, chemical stability, and outstanding biocompatibility. PEEK exhibits an elastic modulus and mechanical properties closely matching those of natural bone, making it an ideal candidate for load-bearing orthopedic applications.^98^ However, the bio-inert surface of pristine PEEK poses challenges for bone integration and cellular adhesion.^99^ Therefore, surface modification of PEEK with bioactive coatings, such as MOFs, is essential to enhance its osteogenic potential and facilitate better biological responses.

Based on this approach, Xiao *et al.*^89^ investigated the effects of surface modification of PEEK implants using a Zn/Mg-MOF74 coating. To facilitate the formation of MOF on the implant surface, PEEK was first treated with polydopamine (PDA), resulting in PEEK-PDA. Subsequently, PEEK-PDA was placed into a hydrothermal reactor containing the necessary precursors (Zn^2+^, Mg^2+^, and 2,5-dihydroxyterephthalic acid) to form a Zn/Mg-MOF74 coating, denoted as PEEK-74. Prior to biological evaluation, PEEK-74 was further loaded with DEX to facilitate bone regeneration, yielding the final material, PEEK-DEX. As expected, both PEEK-74 and PEEK-DEX demonstrated significantly improved antibacterial activity against *E. coli* and *S. aureus* compared to PEEK-PDA and unmodified PEEK, which can be attributed to the combined effects of ion release and drug delivery from the coating. Moreover, *in vivo* studies revealed that PEEK-DEX markedly accelerated new bone formation after 9 days compared to bare PEEK. These findings suggested that MOF-based coatings combined with drug loading on PEEK implants hold the ability to enhance antibacterial performance and promote bone regeneration, offering promising prospects for clinical applications in the treatment of complex bone defects (Fig. 5b).

## 4. Integration of MOFs with inorganic biomaterials for bone tissue engineering

### 4.1. Core–shell structures

Recently, the construction of core**–**shell structures combining MOFs with inorganic components such as calcium phosphate (CaP) or mesoporous silica nanoparticles (MSNs) has attracted significant attention in bone tissue engineering. This design strategy leveraged the advantages of both materials: MOFs offered the controlled-release behavior of drugs and ions, while CaP and MSN provided excellent cell adhesion and promoted bone mineralization. The synergistic integration of these components not only strengthens biological stability but also maximizes bone regeneration outcomes and modulates inflammatory responses, making them promising candidates for advanced bone repair applications (Table 3).

**Table 3:** MOFs incorporating inorganic biomaterials for bone tissue engineering^a^

MOF	Biomaterial	Bioactive	MOF-based biomaterial	Stimuli	Properties	Ref.
**Core–shell structure**						
Mg-MOF-74	MSN	—	Mg-MOF-74@MSN	—	Osteogenesis	122
UiO-66	CaP	CpG, ZOL	ZOL/UiO-66@CpG		Bone-targeted drug delivery and anti-tumor properties	124
ZIF-8	Cu_2−*X*_Se	ICG	ICG/Cu_2−*X*_Se@ZIF-8	NIR	Anti-tumor properties	125
MgGA bioMOF	CaP, MSN	IL4	CaP coated MSN/IL4@MOF	pH	Osteogenesis	120

**MOF modified Ti implant**						
Bio-MOF-1	AHT	—	Bio-MOF-1 modified AHT	—	Osteogenesis and enhanced osseointegration	123
ZIF-8	AHT	—	ZIF-8 modified AHT	—	Osteogenesis and enhanced osseointegration	126
ZIF-8	AHT	—	ZIF-8 modified AHT	—	Osteogenesis and antibacterial properties	127
Ce/Sr-PXBP bioMOF	AHT	—	Ce/Sr-PXBP modified AHT	H_2_O_2_	Osteogenesis, enhanced osseointegration and mitochondria-targeted ability	128
ZIF-67	TNT	OGP	OGP@ZIF-67 modified TNT	—	Osteogenesis, enhanced osseointegration, and antibacterial and anti-inflammatory properties	129
ZIF-8	Ti_6_Al_4_V	RSD	RSD@ZIF-8 modified Ti_6_Al_4_V	—	Biocompatibility and enhanced osseointegration	130
ZIF-8	TNT	Nar	Nar@ZIF-8 modified TNT	pH	Osteogenesis, enhanced osseointegration and antibacterial properties	131
ZIF-8	Ti_6_Al_4_V	Iodine	Iodine@ZIF-8 modified Ti_6_Al_4_V	NIR	Osteogenesis and antibacterial properties	132
Zr-Fc MOF	Ti plate	DOX	DOX@Zr-Fc MOF modified Ti	NIR, H_2_O_2_	Osteogenesis, stimuli-responsive drug release and anti-tumor properties	121

a CaP: calcium phosphate; IL4: Interleukin-4 protein; MSN: mesoporous silica nanoparticle; AHT: alkali-heat treated titanium; ICG: indocyanine green; TNT: titania nanotubes; Nar: naringin; OGP: osteogenic growth peptide; Ti: titanium plates; DOX: doxorubicin; ICA: icariin; BG: bioglass; VAN: vancomycin; RSD: risedronate; CpG: cytosine–phosphate–guanosine; β-TCP: beta-tricalcium phosphate.

For example, Li and co-workers^122^ proposed a core–shell structure of Mg-MOF-74@MSN to control the release of Mg^2+^ ions, which are essential for bone development and regeneration. Specifically, Mg-MOF-74 was easily synthesized through a hydrothermal method. Subsequently, an approximately 40 nm thick MSN shell was coated onto the surface, forming the Mg-MOF-74@MSN system. The MSN shell effectively regulated the release of Mg^2+^ ions, slowing the release rate by approximately 1.4 times compared to pure MOF. This sustained ion release provided a more stable environment that supported bone marrow mesenchymal stem cell (BMSC) proliferation, which increased by over 50% after five days of culture. These findings imply the prospect of utilizing core–shell structures for enhancing bone regeneration in a more controlled and efficient manner.

**Fig. 6:**
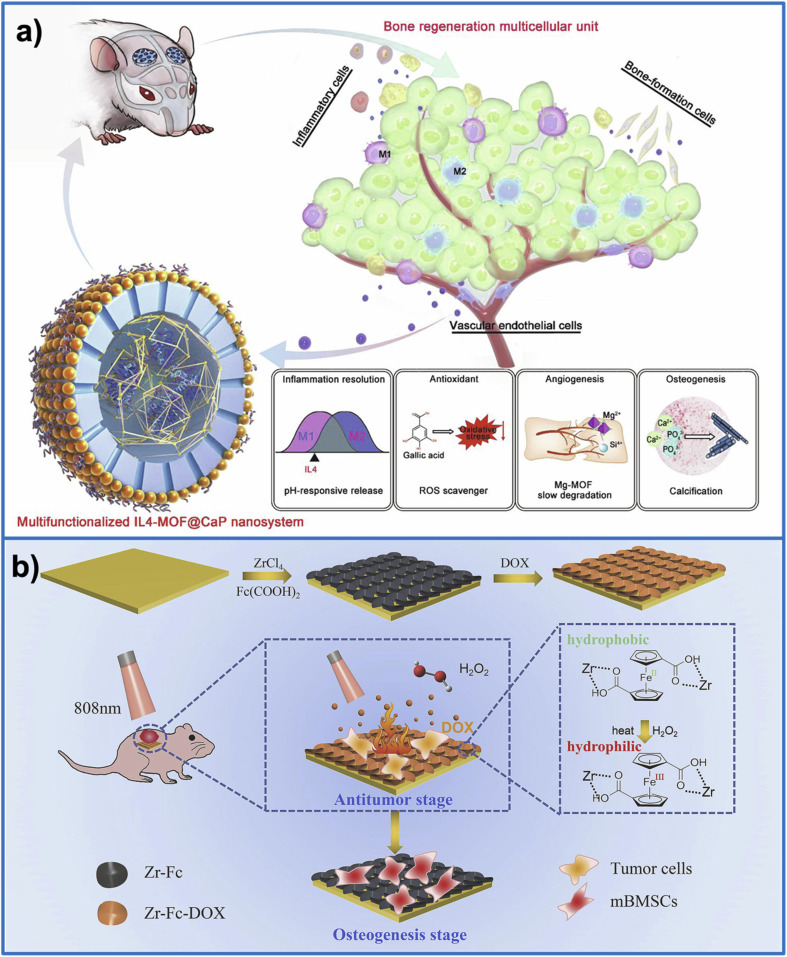
(a) Schematic representation of an IL4-MOF@CaP nanosystem designed to enhance bone regeneration by combining key factors including immunomodulation, antioxidant protection, promotion of angiogenesis, and stimulation of osteogenesis. This figure has been reproduced from ref. 120 with permission from Elsevier, copyright 2020. (b) Schematic illustration of the surface modification of a titanium plate with Zr-Fc MOF and DOX for combined tumor therapy and bone regeneration. Upon dual stimulation with NIR and H_2_O_2_, the Zr-Fc-DOX coated titanium plate not only effectively eliminates osteosarcoma cells but also promotes bone cell adhesion and upregulates osteogenic gene expression to support bone regeneration. This figure has been reproduced from ref. 121 with permission from Elsevier, copyright 2023.

Zheng *et al.*^120^ reported the synthesis of a multifunctional core–shell system based on MgGA bioMOF for applications in bone regeneration. The fabrication of this material involved three main steps. First, Mg-gallate MOF was synthesized to serve as the core structure. Next, an MSN layer was coated onto the Mg-MOF surface, acting as a template to guide and regulate the formation of the outer shell. Finally, a CaP layer was deposited onto the MSNs to create the complete core–shell architecture, referred to as MOF@CaP. Interleukin-4 (IL4) was then incorporated into the system, resulting in IL4-MOF@CaP, to further modulate immune responses and promote tissue regeneration. Both *in vitro* and *in vivo* studies demonstrated that IL4-MOF@CaP enabled controlled release of multiple bioactive factors: magnesium ions to stimulate angiogenesis, gallic acid to scavenge reactive oxygen species, and calcium and phosphate ions to facilitate ECM mineralization. Overall, this multifunctional platform provides a favorable microenvironment for vascularized bone regeneration (Fig. 6a).

### 4.2. MOF-modified Ti-based implants

Titanium (Ti)-based implants are widely used in bone-related biomedical applications due to their excellent mechanical properties, corrosion resistance, and biocompatibility. However, the bioinert nature of Ti implants often hampers direct bone integration and limits their ability to actively support bone regeneration. One of the practical implementations, hydrogel coatings, has been commonly applied to address the issues occurring between the implant and bone tissue. Nonetheless, conventional hydrogels typically lack the capability for controlled and sustained drug delivery, which is crucial for modulating the bone healing environment over time. In this scenario, MOFs, known for their high surface area, tunable porosity, and efficient controlled-release properties, have emerged as suitable candidates for functionalizing Ti implants.

Indeed, Wu *et al.*^123^ reported a successful surface modification strategy for alkali-heat-treated titanium (AHT) implants by directly growing bio-MOF-1, a type of MOF composed of Zn^2+^ and adenine, onto their surfaces. The *in vitro* results demonstrated that the bio-MOF-1@AHT coating significantly triggered osteogenic differentiation of BMSCs by increasing alkaline phosphatase activity, promoting the deposition of ECM minerals, and stimulating the expression of key osteogenesis-related genes. Moreover, in results from an *in vivo* New Zealand white rabbit model, the bio-MOF-1@AHT implants showed superior peri-implant bone integration compared to unmodified AHT.

Yan *et al.*^121^ proposed a surface modification strategy for Ti implants using Zr-Fc MOF loaded with doxorubicin (DOX), aiming to achieve dual functions of tumor therapy and bone regeneration. Specifically, the Ti implant was modified through a hydrothermal process with ZrCl_4_ and 1,1-dicarboxyferrocene, leading to the formation of a Zr-Fc MOF coating on the Ti surface, referred to as Zr-Fc. Subsequently, Zr-Fc was further loaded with DOX to form the Zr-Fc-DOX system. Under the combined effects of NIR irradiation and hydrogen peroxide, Zr-Fc-DOX enabled efficient DOX release, thereby killing human osteosarcoma cells (Saos-2 and 143B). Meanwhile, for BMSCs, Zr-Fc-DOX exhibited superior cell adhesion and significantly upregulated the expression of osteogenic genes (ALP, Col-I, TGF-β, and Runx2) compared to the Ti implant. These results demonstrate the effectiveness of integrating this multifunctional system to provide time-dependent tumor therapy while also promoting bone regeneration (Fig. 6b).

## 5. Integration of MOFs with organic and inorganic biomaterials for bone tissue engineering

Natural bone is an ideal biological composite material, consisting of a well-organized combination of inorganic components (*e.g.*, hydroxyapatite and carbonated apatite) and organic components (*e.g.*, collagen and proteins). Inspired by this structure, the integration of MOFs with both organic and inorganic biomaterials has emerged as a promising strategy to develop multifunctional scaffolds that closely mimic the structure and function of native bone, which has attracted considerable research interest (Table 4).

**Table 4:** MOFs incorporated with organic and inorganic biomaterials for bone tissue engineering^a^

MOF	Organic	Inorganic	Bioactive	MOF-based biomaterial	Stimuli	Properties	Ref.
HKUST-1	PCL and FA	AZ31 Mg alloy	—	FA@HKUST-modified PCL/AZ31 Mg	—	Osteogenesis and anti-corrosive properties	135
ZIF-8	PCL	DCPD	—	ZIF-8 modified PCL/DCPD	—	Osteogenesis	136
MgGA bioMOF	PLGA	DCPD	—	MgGA modified PLGA/DCPD	—	Osteogenesis	137
ZIF-8	PDA and PEI	BCP	—	ZIF-8 modified PDA/PEI/BCP	—	Osteogenesis	138
ZIF-8	PLLA and PDA	HAP	—	HAP/PDA@ZIF-8 modified PLLA scaffold	—	Ion-controlled release and biocompatibility	139
MgGA bioMOF	LCFRPEEK and MACS	HAP	—	HAP@Mg-GA modified MACS/LCFRPEEK	pH	Osteogenesis, angiogenesis and anti-inflammatory properties	133
ZIF-8	SF	Ti implant	DEX	DEX@ZIF-8 modified SF/Ti	—	Osteogenesis and controlled-release drug delivery	140
Mg-MOF-74	SF	Ti6Al4V	ICA	ICA@Mg-MOF-74 modified SF/Ti6Al4V		Osteogenesis, ion-controlled release, anti-inflammatory properties and enhanced osseointegration	141
ZIF-8	CMC	HAP	DEX	DEX@ZIF-8 modified CMC/HAP	—	Controlled-release drug delivery and biocompatibility	134
ZIF-8	PDA, PLGA, and COL	TCP	PDGF	PDA/PDGF@ZIF-8 modified COL/PLGA/TCP	NIR	Osteogenesis and antibacterial properties	142
ZIF-8	COL, Gel, and CS	Ti implant	Levo	Levo@ZIF-8 modified Gel/CS/COL/Ti	pH	Osteogenesis, antibacterial properties and enhanced osseointegration	143
ZIF-8	Gel and PDA	HAP	Cis, BMP-2	*Cis*-BMP-2@ZIF-8 modified Gel/PDA/HAP	pH, H_2_O_2_	Osteogenesis, stimuli-responsive drug delivery and anti-tumor properties	144

a HAP: hydroxyapatite; DCPD: dicalcium phosphate dihydrate; n-HA: nano-hydroxyapatite; Cis: cisplatin; BMP-2: bone morphogenetic protein-2; PEI: polyethyleneimine; BCP: biphasic calcium phosphate; COL: collagen; PDGF: platelet-derived growth factor; Levo: levofloxacin; LCFRPEEK: long carbon fiber-reinforced polyetheretherketone; MACS: methacryloyl chitosan; β-TCP: beta-tricalcium phosphate.

**Fig. 7:**
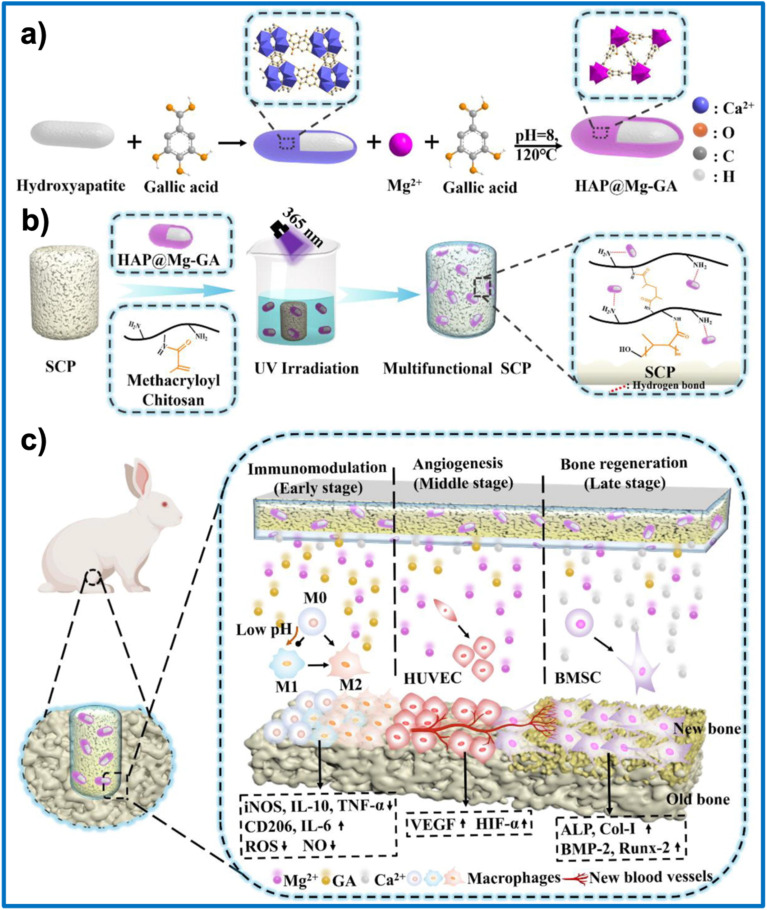
(a) Schematic illustration of the synthesis process of core–shell HAP@Mg-GA nanoparticles, in which hydroxyapatite (HAP) serves as the core and Mg-GA MOF forms the shell. (b) Schematic representation of a multifunctional scaffold (SCP), fabricated by integrating a 3D sulfonated LCFRPEEK scaffold (SCP) with pH-responsive methacryloyl chitosan hydrogel and pro-angiogenic and osteogenic HAP@Mg-GA nanoparticles, under UV-assisted crosslinking. (c) Illustration of the therapeutic performance of the multifunctional SCP scaffold in a rabbit tibial defect model. This figure has been reproduced from ref. 133 with permission from Elsevier, copyright 2023.

Dong *et al.*^133^ developed a multifunctional scaffold (SCP) through the rational integration of inorganic and organic components to enhance bone regeneration outcomes. Specifically, the core framework of this system is a three-dimensional sulfonated long carbon fiber-reinforced polyetheretherketone (LCFRPEEK) scaffold, which exhibits an elastic modulus comparable to that of native bone, thereby improving mechanical strength and tissue integration. To further optimize the local microenvironment, a pH-responsive methacryloyl chitosan hydrogel layer was grafted onto the scaffold surface, providing adaptive responsiveness to pathological conditions. Embedded within this hydrogel are core–shell HAP@Mg-GA nanoparticles, in which the MOF shell functions as an intelligent drug delivery system, enabling the controlled release of magnesium ions and gallic acid to promote angiogenesis and exert antioxidant effects, while the HAP core supplies essential minerals for osteogenesis. As anticipated, both *in vitro* and *in vivo* studies demonstrated that this multifunctional SCP scaffold exhibited superior immunomodulatory properties and promoted neovascularization and bone regeneration compared to each of its components (Fig. 7).

In another study, Sarkar *et al.*^134^ designed a three-dimensional carboxymethyl cellulose-hydroxyapatite (CMC-HA) scaffold modified with DEX@ZIF-8 nanoparticles (CMC-HA/DEX@ZIF-8) as a localized drug delivery system for load-bearing bone applications. In terms of mechanical properties, the CMC-HA/DEX@ZIF-8 composite exhibited a viscoelastic stress–strain behavior under compression, resembling the typical deformation pattern observed in human bone. The composite demonstrated a compressive strength of 16.3 ± 1.57 MPa and an elastic modulus of 0.54 ± 0.073 GPa, both of which fall within the mechanical range of cancellous bone, indicating its suitability for orthopedic applications requiring mechanical support. Regarding drug release, the CMC-HA/DEX@ZIF-8 system provided a sustained and controlled release of DEX *in vitro* over 28 days without showing an initial burst release. Furthermore, *in vitro* studies using MC3T3 osteoblast cells confirmed that the CMC-HA/DEX@ZIF-8 scaffold significantly enhanced both cell proliferation and osteogenic differentiation compared to the unmodified CMC-HA scaffold. These results illustrate the promising potential of developing advanced multifunctional scaffolds capable of simultaneously supporting essential properties such as mechanical integrity and controlled drug delivery for bone regeneration.

## 6. Advances and challenges of MOF and MOF-integrated biomaterial for bone tissue engineering

### 6.1. Molecular building blocks

From a structural perspective, the molecular building blocks of MOFs, comprising metal ions and organic ligands, exhibit high tunability. This intrinsic flexibility enables the design of MOF structures tailored to specific therapeutic objectives in bone tissue engineering. The ability to customize these structural components forms the foundation of MOFs' advantages in biomedical applications.

#### 6.1.1. Metal ions

Based on the data summarized in Tables 1–4, various metal ions have been incorporated into MOF structures for applications in bone tissue engineering. Among them, the most commonly investigated include zinc (*e.g.*, ZIF-8, ZIF-11, ZIF-90, and Bio-MOF-1), magnesium (*e.g.*, Mg-MOF-74, Mg-GA, and Mg-gallate), copper (*e.g.*, HKUST-1 and Cu-TA), zirconium (*e.g.*, UIO-66 and Zr-TCPP), cobalt (*e.g.*, ZIF-67 and Co-TCPP), strontium (*e.g.*, SrPAEM), calcium (*e.g.*, CaPAEM), iron (*e.g.*, MIL-100), cerium (*e.g.*, Ce/Sr-PXBP), and nickel (*e.g.*, Ni-MOF). These metal ions are predominantly essential macro- and trace elements involved in key biological processes such as osteogenic cell differentiation, ECM protein synthesis, and bone tissue mineralization.^145–147^ In addition to their biological functions, certain ions such as Zn^2+^, Cu^2+^, and Co^2+^ exhibit inherent antibacterial properties. Their presence helps reduce the risk of implant-associated infections and fosters a favorable microenvironment for tissue regeneration.^148–150^ Apart from their bioactivity, metal ions significantly influence the physicochemical, mechanical, and biological characteristics of MOF-based systems, including crystal framework stability and biodegradation rates.^134,139^ These features are crucial for modulating drug release kinetics, ensuring material stability under physiological conditions, and promoting integration with native bone tissue.

Despite the promising potential of metal ions incorporated into MOFs for bone regeneration, comprehensive and systematic studies addressing the optimal dosage and safety thresholds of these ions remain scarce. Recognizing the importance of this issue, we reviewed existing literature to gather relevant toxicological reference values. Reported median lethal doses (LD_50_) in rats include magnesium (8 g kg^−1^), zirconium (4.1 g kg^−1^), calcium (1 g kg^−1^), copper (0.025 g kg^−1^), zinc (0.35 g kg^−1^), and iron (0.45 mg kg^−1^). In comparison, the recommended daily intake levels for humans are magnesium (350 mg), zirconium (0.05 mg), calcium (1000 mg), copper (2 mg), zinc (15 mg), and iron (15 mg day^−1^).^151–154^ These findings emphasize the urge for further in-depth toxicological evaluations to assure the safe and effective clinical translation of MOF-based systems in bone tissue engineering.

#### 6.1.2. Organic ligands

Organic ligands, together with metal ions, form the essential molecular building blocks of MOFs. In the context of bone tissue engineering, these ligands are categorized into two main types: biological ligands, which are derived from naturally occurring compounds such as amino acids, nucleobases, carbohydrates, peptides, and natural polyphenols, and synthetic ligands, which include chemically synthesized molecules such as polycarboxylates, imidazolates, and aromatic acids.^155,156^

Biological ligands are generally more favorable in terms of biocompatibility, as they originate from naturally present biomolecules and are more easily recognized and metabolized by living systems. MOFs synthesized using these ligands often exhibit reduced cytotoxicity and can serve as a reservoir to provide bioactive molecules that support bone cell proliferation and differentiation.^63,86,133,157^ However, MOFs built from biological ligands may suffer from lower structural stability and limited porosity in physiological environments, which can hinder their long-term performance.^158,159^

In contrast, synthetic ligands offer better control over pore size, chemical stability, and framework crystallinity. Common examples include terephthalic acid, trimesic acid, 2-methylimidazole, 2,6-naphthalenedicarboxylic acid, 5-aminoisophthalic acid, and gallic acid.^160–162^ These ligands can also be chemically functionalized with groups such as amino, nitro, carboxylate, or methyl to improve their interaction with the biological environment and to regulate drug loading and release behavior.^163–165^

Despite their versatility and growing use in biomedical MOFs, the toxicity and appropriate dosing of organic ligands in bone tissue engineering applications remain poorly studied. To date, no systematic investigations have clearly defined the safe concentration ranges or long-term biological impacts of these ligands when released *in vivo*. Reference data on LD_50_ in rats include the following values: 2-methylimidazole (1.4 g kg^−1^), trimesic acid (8.4 g kg^−1^), terephthalic acid (5 g kg^−1^), 2,6-naphthalenedicarboxylic acid (5 g kg^−1^), 5-aminoisophthalic acid (1.6 g kg^−1^), and gallic acid (5 g kg^−1^).^154,166,167^

### 6.2. Synthesis methods

Overall, the main synthesis methods of MOFs reported for bone tissue engineering include hydrothermal synthesis, solvothermal synthesis, and room-temperature synthesis. Each method presents notable benefits and limitations. Specifically, hydrothermal and solvothermal methods typically facilitate the formation of highly pure and homogeneous MOF crystalline structures. However, these approaches require specific conditions, such as elevated temperatures and pressures, and the use of toxic organic solvents such as DMF. These factors can pose environmental concerns and are difficult to completely remove from the final MOF structure after synthesis.^168,169^ On the other hand, room-temperature synthesis has become increasingly popular due to its energy-saving potential, ease of reaction control, eco-friendly processes, and suitability for large-scale production.^170–173^ Nevertheless, this method is generally limited to specific MOF structures such as ZIF-8 and ZIF-67, using methanol or water as a solvent.

In the context of therapeutic agent loading for bone repair, MOFs can be incorporated with bioactive agents through two main strategies: (i) post-synthetic loading and (ii) one-pot synthesis. Post-synthetic loading enables easy control over drug type and loading content, making it compatible with a variety of bioactive substances.^174,175^ However, this approach may lead to reduced bioactivity or low loading efficiency due to limited surface area for adsorption.^162,165^ In contrast, the one-pot method minimizes processing time, reduces the risk of bioactivity loss, and typically achieves higher loading efficiency.^97,111,143^ Nonetheless, this technique still faces challenges in precisely controlling particle size, morphology, and porosity of the resulting MOFs.^176–178^

The strategy of developing MOF systems coated with inorganic components or targeting agents has been explored as the central topic of numerous studies due to its ability to address issues related to drug or growth factor delivery. These coatings have been shown to not only improve the stability and dispersibility of MOFs, but also to facilitate their targeting capabilities and controlled drug release, thereby contributing to improved bone tissue regeneration outcomes.^102,103,124^ However, these advancements also present certain limitations. The addition of inorganic coatings or biological targeting agents can complicate the synthesis process, making it challenging to precisely control the thickness and uniformity of the coating layers.

On the other hand, when integrating MOFs with inorganic, organic, or hybrid biomaterials, MOFs are commonly anchored *via* direct growth on the surface of the base materials, typically involving pre-formed implants such as Ti alloys, PEEK, or fibers. In this approach, the base materials are often immersed in a solution containing metal precursors and organic linkers to stimulate MOF growth directly on the surface. During this step, additional bioactive agents can be loaded into the MOF structures post-growth. This method offers the advantages of time efficiency and procedural simplicity; however, the adhesion strength of MOFs to the base material may not be as strong as that achieved through direct assembling techniques.^126,127,131^

The direct assembling method is commonly applied when MOFs or MOFs loaded with bioactive agents are incorporated into biological systems, most notably hydrogels. This approach allows precise control over the ratio of components and is well-suited for shaping gel-based systems.^105,109^ Nonetheless, a key challenge of this method lies in ensuring the homogeneous distribution of MOF particles within the hydrogel network, as aggregation or sedimentation may compromise the material's performance.

### 6.3. Physicochemical and mechanical properties

One of the most prominent advantages of MOFs lies in their remarkable ability to flexibly control particle size, ranging from nanometers to micrometers, thereby allowing for adaptation to a variety of biomedical applications. In particular, particles smaller than 200 nm offer substantial benefits in targeted and controlled drug delivery systems, especially in the treatment of bone metastases. MOF nanoparticles at this scale are capable of improving the enhanced permeability and retention effect, which facilitates their preferential accumulation in damaged tissues or tumors while reducing the likelihood of rapid clearance from systemic circulation.^101,124^

Moreover, MOFs possess an impressively high specific surface area, which facilitates the loading of substantial amounts of therapeutic agents, such as anticancer drugs and growth factors.^114,115,178^ The pore size and shape of MOFs can be finely tuned by alternating the use of metal ions or organic ligands, thereby optimizing their capacity for adsorption and the controlled release of bioactive molecules that are required for therapeutic applications.^120,130^ Additionally, the integration of MOFs into various material systems, including hydrogels, electrospun fibers, and three-dimensional scaffolds, significantly augments the porosity and surface area of these materials. This creates a favorable microenvironment that facilitates the infiltration of cells, nutrients, and growth factors, thus supporting efficient bone tissue regeneration.^134,144^

Despite the noteworthy advantages of MOF-based materials in bone tissue engineering, several limitations should be taken into consideration. Although particle size can be effectively controlled during synthesis, achieving homogeneous dispersion of MOF particles within the host matrix remains challenging. This issue is especially pronounced in soft materials such as hydrogels, where poor dispersion may lead to particle agglomeration and inconsistent mechanical properties within the composite material. In addition, while many MOFs exhibit good chemical and thermal stability, some structures are prone to premature degradation under physiological conditions. This degradation can result in the uncontrolled release of metal ions or therapeutic agents, potentially diminishing treatment efficacy and increasing the risk of cytotoxicity. Furthermore, although the incorporation of MOFs can strengthen the mechanical strength of biomaterials, the level of reinforcement achieved is often lower than that provided by conventional materials such as bio-ceramics or metals. This shortcoming restricts the application of MOF-based composites in scenarios that require the repair of high-load-bearing bone defects.

### 6.4. Biological properties

Biological performance represents an essential prerequisite for the clinical translation of MOF-based materials in bone tissue engineering. In addition to favorable biocompatibility and biodegradability, a comprehensive understanding of the underlying molecular and cellular mechanisms is compulsory for improving therapeutic efficacy and ensuring biosafety.

At the cellular level, numerous studies have demonstrated that MOFs can directly interact with bone-associated cells, including osteoblasts, osteoclasts, mesenchymal stem cells, and endothelial cells. MOFs are capable of promoting osteogenic differentiation by upregulating bone-specific markers such as ALP, Ocn, and Runx2. They also activate essential signaling cascades, including the PI3K/AKT-HIF-1α, PI3K/AKT, TGF-β/BMP, MAPK, and calcium signaling pathways.^58,70^ Concurrently, certain MOFs demonstrated effective angiogenic properties by stimulating the expression of vascular endothelial growth factor and other pro-angiogenic mediators in endothelial cells, thus contributing to neovascularization and bone regeneration.^58,70,82^ Furthermore, the controlled release of metal ions such as zinc, strontium, calcium, and magnesium plays a dual role: supporting bone matrix mineralization while modulating osteoclast-mediated bone resorption.^137,138,140^ These molecular-level interactions indicate that MOFs act not only as passive drug carriers but also as bioactive agents that participate in cell signaling regulation, ECM remodeling, and immunomodulation.

In terms of biocompatibility, extensive *in vitro* studies have confirmed that MOFs such as ZIF-8 (up to 100 μg mL^−1^) and Mg-MOF-74 (up to 1000 μg mL^−1^) exhibit negligible cytotoxicity toward bone-relevant cells, including rBMSCs, MG-63, and RAW264.7.^69,101,102,122^ Furthermore, bioMOFs composed of endogenous metal ions and biologically active ligands (*e.g.*, adenine and gallic acid) demonstrate low cytotoxicity, favorable cellular uptake, and enhanced osteogenic potential.^63,123,133^ When used as drug delivery systems, MOFs have shown the ability to improve the therapeutic performance of agents such as vancomycin, dexamethasone, and simvastatin through targeted and controlled release mechanisms.^78,87,128^ Furthermore, MOFs and biomaterials derived from them have achieved expected results such as stimulating cell proliferation and differentiation in *in vivo* models with bone damage (*e.g.*, rat, mouse, and rabbit).^86,126,138,143^

Regarding biodegradability, most MOFs possess inherent degradability in physiological environments. This behavior primarily stems from the relatively weak coordination bonds between metal ions and organic ligands, which are susceptible to dissociation under biological conditions, particularly in complex microenvironments such as bone implantation sites or metastatic bone tissues. Although the degradation of MOFs can be beneficial for releasing therapeutic ions and bioactive compounds, uncontrolled or rapid degradation may lead to excessive ion release, posing potential risks of cytotoxicity and inflammatory responses.^117,132,144^

To address this issue, various functionalization strategies have been elaborated using inorganic and organic modifiers to regulate MOF stability and modulate ion release kinetics safely and therapeutically. A representative example is ZIF-8, one of the most widely used MOFs, whose stability has been significantly enhanced through biomaterial integration. Specifically, functionalization with gelatin and chitin has extended its structural integrity to approximately 10 days under physiological conditions.^143^ Incorporation with polycaprolactone and gelatin has prolonged its degradation to 21 days,^115^ while coating with polydopamine and hydroxyapatite has further increased its stability up to 29 days.^139^ These findings demonstrated the feasibility of tailoring the biodegradation profile of MOFs to meet specific therapeutic needs, providing a solid foundation for the development of clinically applicable, bone-regenerative MOF-based biomaterials.

## 7. Perspectives and future recommendations for MOFs and MOF-based biomaterials in bone tissue engineering

There is a growing complexity in bone-related issues, encompassing various factors such as bone fractures, bone cancer, bone degeneration, bone infections, and other subjective and objective elements. Conversely, investigations into the application of MOFs in the domain of bone regeneration have made substantial advancements and are expected to demonstrate robust growth in recent times. Based on this idea, it is evident that MOFs possess the potential to emerge as a promising biomedical material alternative for addressing bone-related issues. Below, we will provide our perspectives on the future potential of MOFs in bone engineering, from a medical, technological, and economic perspective. Our objective is to offer researchers significant recommendations regarding the properties and potential uses of MOF materials.

### 7.1. Medical perspective

Based on the analysis of Tables 1–4, it is evident that current studies in bone tissue engineering predominantly focus on Zn-based MOFs, indicating a rather unidirectional research trend. To broaden the scope of application and fully exploit the potential of MOF materials in this field, it is essential to promote investigations into other MOF systems based on metals that play critical roles in bone metabolism and regeneration, such as calcium, strontium, magnesium, and copper. Moreover, the development of bioMOFs should gain more attention due to their superior biocompatibility and biodegradability compared to conventional MOFs, which could enhance their performance in bone-related applications. Furthermore, to ensure the safe and effective clinical translation of MOF-based systems for bone regeneration, future research should systematically assess the dose–response relationship, long-term toxicity, and biological fate of MOFs within physiological environments. These efforts will be crucial in guiding the rational design of MOFs and MOF-based composites for bone tissue engineering applications.

### 7.2. Technical perspective

Advanced techniques such as electrospinning and three-dimensional printing have been employed to integrate MOFs into various material systems, including inorganic, organic, and hybrid inorganic–organic composites for bone tissue engineering applications. However, most current studies remain focused on optimizing individual fabrication parameters through an empirical trial-and-error approach by adjusting factors such as mixing ratios, temperature, pressure, and reaction time. This experience-based methodology lacks a systematic framework for material design, which may negatively impact research efficiency, lead to excessive resource consumption, and present challenges in establishing quantitative structure–property relationships, ultimately hindering large-scale implementation.

To overcome these challenges, the implementation of advanced technological tools such as artificial intelligence, data science, computational modeling, and machine learning in the design and development of MOF-based composites has become increasingly essential. These technologies can facilitate accurate prediction of material properties, optimize compositions and synthesis conditions, thereby significantly reducing experimental workload, accelerating development timelines, and improving cost-effectiveness.

Nonetheless, it is important to emphasize that this approach is inherently complex and requires close interdisciplinary collaboration across materials science, computational engineering, chemistry, biology, and data science. Only through strong interdisciplinary integration can the intelligent, efficient, and application-driven design of MOF-based composites be successfully achieved. This collaborative approach is particularly crucial for bone tissue engineering, where both structural integrity and biological functionality must be precisely engineered to meet clinical requirements.

### 7.3. Economic perspective

Most current studies on MOF materials and MOF-based composites for bone tissue engineering have primarily focused on evaluating therapeutic performance, such as tissue regeneration capacity, drug delivery efficiency, or biocompatibility. However, the aspects of production costs and economic feasibility have not yet been systematically addressed. This represents a significant limitation, as production cost directly influences the scalability of materials and plays a critical role in determining their future potential for commercialization. Therefore, introducing economic evaluation into MOF-related studies is imperative to ensure practical applicability and clear translational direction for biomedical products.

As mentioned above, the application of advanced tools such as artificial intelligence, computational modeling, and machine learning can support the optimization of material structures and synthesis conditions. This approach helps save time, reduce experimental costs, and improve overall research efficiency, thereby offering a practical solution for developing MOF-based composites more cost-effectively and systematically.

Notably, the field of MOFs has experienced a rapid evolution in recent years, with more than 90 000 distinct structures synthesized and reported, demonstrating remarkable structural and functional diversity. Nevertheless, only a small fraction of these MOFs have been commercialized into specific products, Basolite® Z1200 (ZIF-8), Basolite® A100 [MIL53(Al)], Basolite® C300 (HKUST-1), and Basolite® F300 (Fe-BTC). This highlights the vast untapped potential for the transfer and commercialization of MOF-based products, especially in the field of bone tissue engineering, where the demand for high-performance materials continues to grow. Such potential also serves as a driving force for future application-oriented and market-driven research.

## 8. Conclusion

This review has provided a comprehensive overview of the diverse applications of MOFs in the field of bone tissue engineering. The discussion spans from pristine MOF structures to composite systems incorporating MOFs with organic, inorganic, and hybrid biomaterials. The integration of MOFs into platforms such as biomedical implants, hydrogels, electrospun fibers, biocements, and three-dimensional scaffolds has demonstrated significant potential in modulating drug release and enhancing tissue regeneration. Apart from summarizing the current advancements, this review has critically elucidated the remaining challenges associated with the chemical composition, biological performance, and synthesis strategies of MOF-based systems. Building upon these insights, we outline key future directions to facilitate the rational design and effective utilization of multidimensional MOFs in regenerative medicine, particularly for the treatment of bone-related disorders. Despite the remarkable potential of MOFs and MOF-based composites in bone tissue engineering, their clinical translation remains hindered by several obstacles, including concerns regarding toxicity, biological stability, drug release control, and production costs. Addressing these limitations requires a more systematic and interdisciplinary research approach that bridges materials science, biology, computational modeling, and biomedical engineering.

## Author contributions

Luan Minh Nguyen contributed to conceptualization, investigation, data curation, methodology, writing – original draft, and writing – review & editing. Yufeng Wang contributed to investigation, data curation, and writing – review & editing. Giao ThuyQuynh Vu contributed to investigation, data curation, and writing – original draft. Qui Thanh Hoai Ta contributed to methodology and investigation. Dieu Linh Tran contributed to methodology and investigation. Ngoc Hoi Nguyen contributed to methodology and investigation. Thuan Van Tran contributed to data curation, investigation, and writing – review & editing. Chao Zhang contributed to writing – review & editing, supervision, and project administration. Dai Hai Nguyen contributed to writing – review & editing, supervision, and project administration.

## Conflicts of interest

The authors declare that they have no known competing financial interests or personal relationships that could have appeared to influence the work reported in this paper.

## Data Availability

Data will be made available on request.
